# Evaluation of estrogenic, antiestrogenic and genotoxic activity of nemorosone, the major compound found in brown Cuban propolis

**DOI:** 10.1186/1472-6882-13-201

**Published:** 2013-07-31

**Authors:** Mariana S Camargo, Aline M Prieto, Flavia A Resende, Paula K Boldrin, Cassia RP Cardoso, Mariana F Fernández, José Manuel Molina-Molina, Nicolás Olea, Wagner Vilegas, Osmany Cuesta-Rubio, Eliana A Varanda

**Affiliations:** 1 UNESP- Univ. Estadual Paulista, Faculty of Pharmaceutical Sciencies of Araraquara-Department of Biological Sciences, Rodovia Araraquara-Jaú, Km 1, 14801-902 Araraquara, São Paulo, Brazil; 2 School of Pharmacy, University of Granada, Granada, Spain; 3 UNESP- Univ. Estadual Paulista, Campus do Litoral Paulista, Unidade São Vicente, CEP 11330-900 São Vicente, São Paulo, Brazil; 4 Instituto de Farmacia y Alimentos (IFAL), Universidad de La Habana, La Habana, Cuba

**Keywords:** Nemorosone, *Clusia rosea*, Propolis, Estrogenicity, Genotoxicity

## Abstract

**Background:**

Brown propolis is the major type of propolis found in Cuba; its principal component is nemorosone, the major constituent of *Clusia rosea* floral resins. Nemorosone has received increasing attention due to its strong *in vitro* anti-cancer action. The citotoxicity of nemorosone in several human cancer cell lines has been reported and correlated to the direct action it has on the estrogen receptor (ER). Breast cancer can be treated with agents that target estrogen-mediated signaling, such as antiestrogens. Phytoestrogen can mimic or modulate the actions of endogenous estrogens and the treatment of breast cancer with phytoestrogens may be a valid strategy, since they have shown anti-cancer activity.

**Methods:**

The aim of the present investigation was to assess the capacity of nemorosone to interact with ERs, by Recombinant Yeast Assay (RYA) and E-screen assays, and to determine by comet assay, if the compound causes DNA-damaging in tumoral and non-tumoral breast cells.

**Results:**

Nemorosone did not present estrogenic activity, however, it inhibited the 17-β-estradiol (E_2_) action when either of both methods was used, showing their antiestrogenicity. The DNA damage induced by the benzophenone in cancer and normal breast cells presented negative results.

**Conclusion:**

These findings suggest that nemorosone may have therapeutic application in the treatment of breast cancer.

## Background

The *Clusia* genus includes about 300 species that occur throughout the interneotropical realm, from southern USA and Mexico to southern Brazil and Bolivia [1]. Many *Clusia* species produce resiniferous waxes in staminate, and/or pistillate flowers, providing material for honey bees nest construction [2]. Production of these resins is a significant pollinator attractant [1].

The bees collect floral resins from the *Clusia* genus in order to produce propolis [2,3] that is widely used in traditional medicine and is reported to have a broad spectrum of pharmacological properties [4]. Propolis has recently gained popularity as a health food supplement and is used extensively in foods and beverages in various parts of the world; this compound has been the subject of many studies due to its antibacterial, antifungal, antiviral and hepatoprotective activity. Water or alcohol-soluble propolis and its many compounds have been used in the treatment of inflammation, for immuno-stimulation and as an anticancer agent [5]. Its chemical composition is qualitatively and quantitatively variable, depending on the vegetation in the area from which it was collected [6].

**Figure 1:**
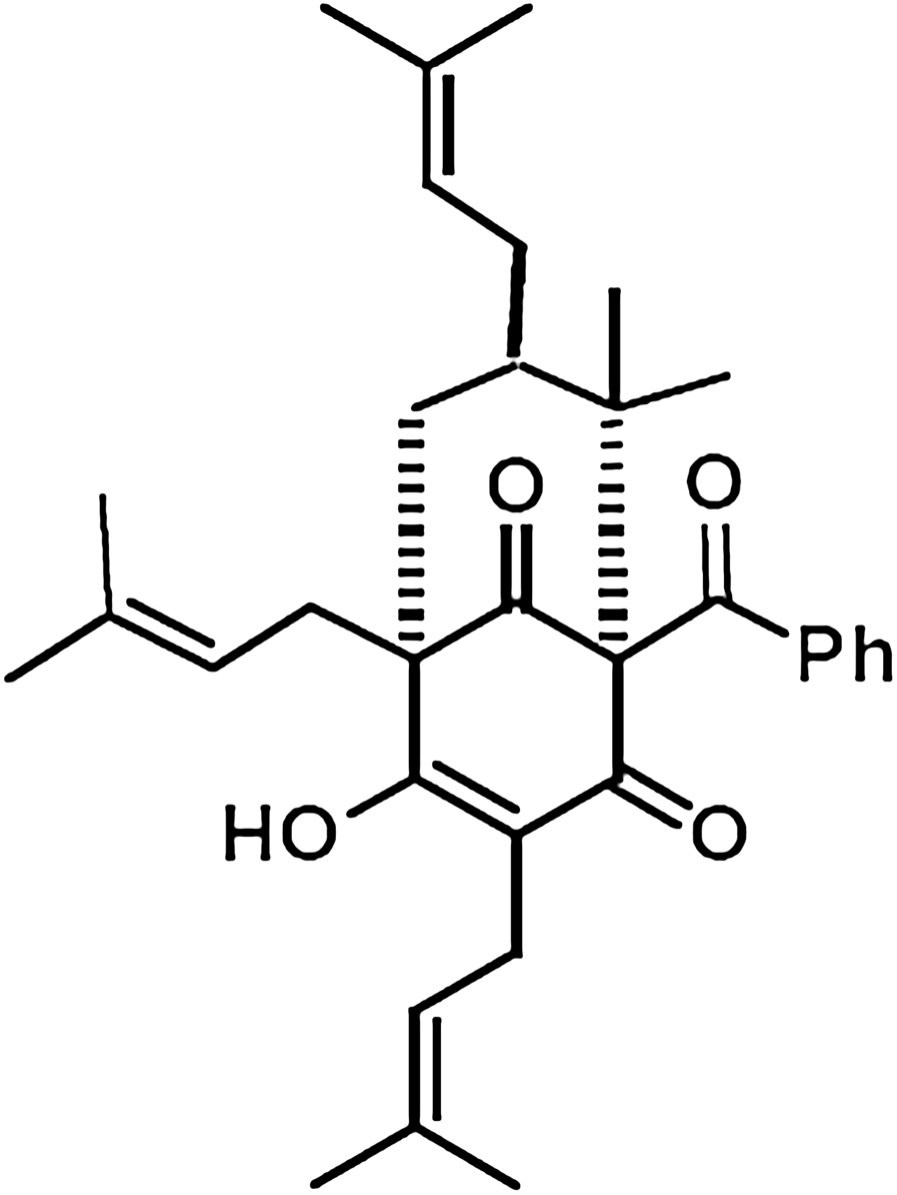
Nemorosone.

Brown propolis is the major type of propolis in Cuba; its chemical composition is exclusive and the principal component is nemorosone (Figure 1) [7], which is the major constituent of *Clusia rosea* floral resins [8,9].

Nemorosone is responsible for the antimicrobial activity of *Clusia* spp. resin and propolis [10]. This compound is a natural-occurring polycyclic polyprenylated acylphloroglucinols and has received increasing attention due to its strong *in vitro* anti-cancer action; [11,12]. The cytotoxicity of nemorosone to several human cancer cell lines has been reported [2] and it has been shown that an ethanol extract of brown cuban propolis exerts a significant cytotoxic activity against breast cancer cells (MCF-7), which is correlated to a direct action on the estrogen receptor (ER) [7,9].

Estrogens have been recognized as the main hormones that stimulate the growth and development of breast cancers. Their activities are mediated by two main isoforms of intracellular ERs: ER*α* and ER*β*[13,14]. ER*α* is expressed in about 75% of diagnosed breast tumors [7] and can thus be treated with agents targeting estrogen-mediated signaling, such as antiestrogens [15,16] . The introduction of adjuvant systemic therapy led to a significant improvement in post-surgical survival and a reduction in disease relapse, especially in women with early breast cancer and with positive ER tumors, who may receive endocrine therapy alone or in combination with cytotoxic therapy [17].

Phytoestrogen is any plant substance or metabolite that induces biological responses in vertebrates and can mimic or modulate the actions of endogenous estrogens [18]. Breast cancer treatment done with phytoestrogens may be a valid strategy; they are widely distributed in the diet and herbs and have shown anti-cancer activity via mechanisms, which include ER modulation, aromatase inhibition and anti-angiogenesis [19].

Recently, in previous studies, nemorosone presented no estrogenic activity in the RYA and no mutagenic activity in the Ames Test. On the other hand, the compound presented antimutagenic activity, reducing DNA damages induced by the antitumoral mytomicin C [20]. In the order to complement the above results, in view of the outstanding antiproliferative potential of nemorosone and the small number of studies published on its mechanisms of action, the aim of the present investigation was to assess the possible capacity of benzophenone to interact with ERs, by RYA and E-screen assays, and to determine, by comet assay, if the compound causes DNA-damage in tumoral and non-tumoral breast cells.

## Methods

### Plant material

Flowers of *Clusia rosea* Jacq. (Gutiferae), were collected in Havana (Cuba) in September 2009 and identified by Dr. Victor Fuentes Fiallo. A voucher specimen (No. 9576) was deposited in the Herbarium of La Estacion Experimental de Plantas Medicinales de Guira de Melena. Nemorosone was extracted from the floral resin of *C. rosea* and isolated as previously reported by Cuesta-Rubio *et al*. [8].

Nemorosone was crystallized from floral resin of *C. rosea*, employing a mixture of EtOH-H_2_O. The product was subjected to vacuum liquid chromatography (VLC) on silica gel with C_6_H_12_-EtOAc (3:1) to purify it to homogeneity. It was identified by 1D- and 2D-NMR experiments, and its purity was verified by HPLC-DAD and HPLC-MS. The structure of nemorosone (C_33_H_42_O_4_) is a mixture of tautomers: 1-benzoyl-4-hydroxy-8,8-dimethyl-3,5,7-tris(3-methyl-2-butenyl)-bicyclo-[3.3.1]-non-3-ene-2,9-dione and 1-benzoyl-2-hydroxy-8,8-dimethyl-3,5,7-tris(3-methyl-2-butenyl)-bicyclo-[3.3.1]-non-2-ene-4,9-dione. The crystallization process yielded white crystals (EtOH/H_2_O); mp 66–68°C; ESI-MS (positive mode), *m*/*z* 503 [M + H]^+^. ^1^H and ^13^C NMR data were consistent with a previous report [8].

### Determination of antiestrogenic activity by Recombinant yeast assay (RYA)

Yeast strain BY4741 (MATa *ura3Δ0 leu2Δ0 his3Δ1 met15Δ0*) from Euroscarf (Frankfurt, Germany), kindly provided by Dr. Benjamin Piña (CSIC-Barcelona-Spain) was transformed with plasmids pH5HE0 and pVitBX2, as described elsewhere [21]. The expression plasmid pH5HE0 contains the HE0 human ER [22] cloned into the constitutive yeast vector expression pAAH5. The reporter plasmid p VitBX2 contains two copies of the pseudopalindromic estrogen responsive element ERE2 from *Xenopus laevis* vitellogenin B1gene (5′AGTCACTGTGACC-3′) inserted into the unique *Kpn*I site of pSFLΔ-178 k [23]. In brief, transformed yeast cells were first grown overnight in non-selective medium (YPD) at 30°C. Next, the growing cells were transferred to minimal medium (6.7 g/L yeast nitrogen base without amino acids, Difco, Basel, Switzerland; 20 g/L glucose, supplemented with 0.1 g/L of prototrophic markers as required) and incubated overnight at 30°C. The final culture was adjusted to an optical density (OD) of 0.1 and distributed in a siliconized 96-well polypropylene microtiter plate. Aliquots of 10 μL nemorosone solutions in dimethyl sulfoxide (DMSO) at a concentration of 40 μg/well were added to 90 μL of yeast culture. These initial solutions were used for subsequent serial dilutions (1:10, 1:30, 1:90, 1:270 and 1:810). 17-β-estradiol (E_2_) at a final concentration of 10 nM was added to all these wells.

Positive controls were made by adding E_2_ (10 nM) to the yeast culture without the presence of nemorosone. Negative controls were made with pure cultures and by adding 10 μL of DMSO on the yeast culture. A solvent control was made by adding 10 μL of DMSO to the yeast culture with E_2_. The cytotoxicity was assayed on separate YPD agar plates on which the following were spread: pure yeast culture; yeast culture+E_2_; yeast culture+E_2_+nemorosone in the major concentration (40 μg/well); yeast culture+E_2_+DMSO. After 24hours, tests growth was compared to the pure yeast culture growth (100% viability) and the nemorosone concentration which permitted 80% viability, was used in the subsequent assays.

The 96-well plates were incubated for 6 h at 30°C with mild shaking. After incubation, 50 μL of the yeast cell lysis reagent Y-PER^TM^ (PierceTM, Rockford, IL, USA) was added to each well and further incubated at 30°C for 30 min. Finally, 50 μL of assay buffer was added to the lysed cells. The assay buffer was prepared by mixing 100 mL Z-buffer, 1 mL Triton X-100, 1 mL 10% SDS, 70 μL 2-mercaptoethanol and 21 mg 4-methyl umbelliferyl β- D-galactoside. Z-Buffer (pH7.0) is a mix of: 60 mM Na_2_HPO_4_ , 40 mM NaH2PO4, 10 mM KCl and 1 mM MgSO_4_. Plates were read in a spectrofluorometer (Tecan SpectraFluor Plus), set at 355 nm excitation and with 460 nm emission, for 20 minutes, after brief centrifugation The fluorescence induced by E_2_ (positive control) was considered 100% of the estrogenic activity. The entire assay was performed on three replicates.

### Cell lines

#### MCF-7 BUS

Human MCF-7 BUS breast cancer cells were kindly provided by Laboratory of Medical Investigations (Department of Environmental Medicine; University of Granada, Granada, Spain) and were cultured in Dulbecco’s Modified Eagle Medium (DMEM) with 15 mg/L phenol red, 10% fetal calf serum (FCS), 2% of 200 mM l-glutamine, 2% of 1 M HEPES buffer, 1% of 100 mM sodium pyruvate and 1% of 10 mg/mL penicillin–streptomycin, at 37°C in an atmosphere of 5% CO_2_ and 95% air at saturating humidity.

#### MCF10A

Human MCF10A non-tumorigenic breast cells were kindly provided by Dr. Maria Mitzi Brentani of the Radiology Department Oncology Laboratory (Faculty of Medicine, São Paulo University, São Paulo, Brazil) and were cultured in DMEM/Ham’s F-12 medium (Gibco, Invitrogen) supplemented with 100 ng/mL cholera toxin (Calbiochem, La Jolla, CA), 0.01 mg/mL insulin (Sigma, Saint Louis, MO), 500 ng/mL hydrocortisone (Sigma), 20 ng/mL epidermal growth factor (Sigma) and 5% horse serum (Gibco, Invitrogen) at 37°C in an atmosphere of 5% CO_2_ and 95% air under saturating humidity.

#### Determination of estrogenic and antiestrogenic activity by E-screen assay

The simple and sensitive E-screen cell proliferation assay was performed with the human MCF-7 BUS breast cancer cell line. These cells express high levels of ERα and are considered to be the most sensitive line in existence [24]. Growth stimulation of the MCF-7 BUS by compounds, was measured as described in [25], with modifications by [26].

Subconfluent MCF-7 BUS cells were trypsinized and seeded in 24-well plates at an initial density of 2 × 10^4^ cells per well in DMEM with 10% (v/v) FCS (1 mL/well). After cell adhesion to well bottoms (24 h of incubation; 37°C, 5% CO_2_), the cells were washed with phosphate buffered saline (PBS) and the culture medium was changed to DMEM supplemented with 10% of charcoal dextran-stripped (steroid-free) fetal calf serum (FCS). The steroid-free experimental medium thus consisted of phenol-red-free DMEM supplemented with 10% of stripped FCS, 2% of 200 mM l-glutamine, 2% of 1 M HEPES buffer, 1% of 100 mM sodium pyruvate and 1% of 10 mg/mL penicillin-streptomycin.

The positive and negative controls used were 10^-8^M E_2_ and steroid-free experimental medium, respectively. There was also a solvent control (0.01% DMSO, which is the maximum concentration of solvent used in test in steroid-free experimental medium) and a medium control (10% FCS in DMEM).

Nemorosone was added to the experimental medium at a range of concentrations from 10^-9^ to 10^-5^ M. The concentrations were selected on the basis of a preliminary toxicity test employing the sulforhodamine B (SRB) assay [27]. Each experiment was performed three times on triplicate samples.

The assay was terminated after 144 hours of incubation by removing the medium from the wells and then the SRB assay was conducted.

For antiestrogenicity tests, 10^-8^ M of E_2_ was added to the wells with the nemorosone, before incubation.

The estrogenic activity results were expressed as mean ± standard deviation of the proliferative effect (PE) and relative proliferative effect (RPE). The PE represents the proliferation induced by the test substance in MCF-7 BUS cells. This parameter is calculated as described by [24] being the ratio of the highest cell number achieved with E_2_ or the test sample to the cell number in the negative control:

PE=absorbancecompound/absorbancenegativecontrol

The RPE compares the maximal proliferation induced by E_2_ with that induced by a test compound

$$\mathrm{RPE}\%=\left((\mathrm{PE}‒1)\;\text{sample}/(\mathrm{PE}‒1)\;E2\right)\times 100$$

The RPE classifies E_2_ total agonists when they induce a relative proliferation between 80% and 100%. Partial and weak agonists induce a cell proliferation from 25% to 80%, and from 10% to 25%, respectively [28]. For the calculation of these parameters, Excel (Microsoft, NY, USA) formulae and functions were used.

#### Comet assay

For the comet assay, MCF-7 BUS and MCF10A cells were seeded in 24-well plates at an initial concentration of 2 x 10^4^ cells per well and treated with nemorosone at a range of concentrations from 10^-9^ to 10^-5^ M, for 3 and 24 hours. The positive and negative controls used were methyl methanesulfonate 2 μM and experimental medium, respectively. There was also a solvent control (0.01% DMSO, which is the maximum concentration of solvent used in test).

After resuspending the cells, the following steps of the assay were carried out [29]: (I) coating of microscope slides with normal-melting-point agarose; (II) transfer of the treated cell suspension to a microcentrifuge tube; (III) suspension of 20 μL of cells in 100 μL of a 0.5% low-melting agarose solution at 37°C; (IV) pousing of 100 μL of this final suspension on the slides, which had been kept in a freezer for 5 min. The alkaline version of the comet assay (single cell gel electrophoresis) was performed as described by Singh et al. [30]. Briefly, 100 μL of cells were taken, homogenized with low-melting point agarose, spread on a microscope slide pre-coated with normal-melting-point agarose and covered with a coverslip. After 5 min at 4°C, the coverslip was removed from the slides and they were immersed in cold lysing solution (2.4 M NaCl; 100 mM ethylenediaminetetraacetic acid (EDTA); 10mM Tris, 10% dimethylsulfoxide and 1% Triton-X, pH 10) for 24 h. After lysis, the slides were placed in an electrophoresis chamber, covered with electrophoresis buffer (300 mM NaOH plus 1 mM EDTA, pH >13) and left for 20 min for the DNA to unwind. The electrophoresis ran for 20 min (25 V and 300 mA), after which the slides were submerged for 15 min in a neutralization buffer (0.4 M Tris–HCl, pH7.5) and fixed in 96% ethanol for 5 min. Duplicate slides were stained with ethidium bromide and 50 cells were screened per sample in a fluorescent microscope (OLYMPUS® XM10) equipped with an excitation filter of 515–560 nm, a barrier filter of 590 nm and a×40 objective. The level of DNA damage was assessed by an image analysis system (TriTek CometScore™ 1.5, 2006) and the percentage of DNA in the tail (%DNA) and *Olive Tail Moment* (OTM) were obtained.

#### Statistical analysis

In the RYA assay, the fluorescence in the presence of nemorosone was analyzed with GraphPad Prism 5.0 (GraphPad Software Inc., San Diego, CA) using the ANOVA test followed by the Dunnet post-test in order to detect significant inhibition of E_2_ induced fluorescence after nemorosone treatment. The same statistical analysis was used to evaluate the antiestrogenic activity by E-screen assay, in order to detect significant inhibition of E_2_ induced proliferation on MCF-7 BUS (100% proliferation) by the nemorosone treatment. The comet assay were analyzed with the non-parametric Kruskal-Wallis test followed by the Dunn post-test [31] to detect significant DNA damage induced by nemorosone in relation to negative control.

## Results and discussion

A series of *in vitro* assays have been developed in order to detect predominant mechanism of action of potential estrogens. Most of these assays fall into one of three categories: a) ER competitive binding assays that measure the binding affinity of a chemical for the ER; b) reporter gene assays that measure ER binding-dependent transcriptional and translational activity and c) cell proliferation assays that measure the rate of increase in the number of target cells during the exponential phase of proliferation [32].

The recombinant yeast cells were designed and engineered for exquisite sensitivity to estrogens [33]; overexpression of human ER, high amplitude frog vitellogenin estrogen response elements, and their tandem arrangement in the reporter plasmid all serve to amplify β-galactosidase production and hence sensitivity towards E_2_[34]. This assay involves not only agonist binding to the receptor but also receptor-mediated transcription. It can to detect the *in vitro* transcriptional potential of compounds and confirm specificity in the ER response with the typical estrogenic compounds and E_2_[35].

**Figure 2:**
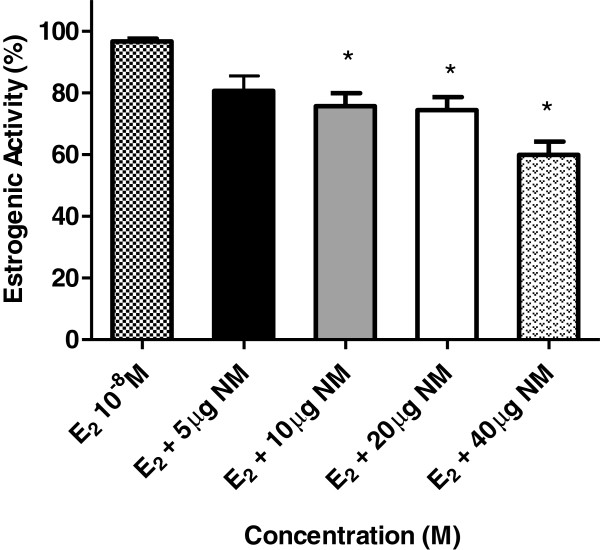
**Dose/response plot for various nemorosone (NM) dilutions in presence of 17 β-estradiol (E**_**2 **_**10**^**-8**^**M) and positive control (100% estrogenic activity) in the RYA system.** The graph shows the β-galactosidase activity in percentage (estrogenic activity) for each nemorosone dilution, from three experiments performed in triplicate. These results are expressed as the mean±SD of three separate experiments. Values significantly different from the E_2_ are indicated by an asterisk (*p<0.05 by Student’s t-tests).

Our results show that nemorosone reduced significantly the estrogenic activity induced by E_2_ (Figure 2). Antagonist activity of nemorosone was detected at 3 concentrations by this assay. The estrogenic activity of E_2_ was reduced in 31%, 34% and 42% by nemorosone at 10, 20 and 40 μg/well, respectively.

The ERs agonist activity of nemorosone by RYA was already evaluated by Camargo et al. [20] and the results showed an absence of estrogenicity by its benzophenone. These results show that nemorosone does not have the capacity of binding to the ER and mediating the transcription in an agonist away (causing the same effects of the estradiol), but also reduces the capacity of 17 E_2_ to exercise this function.

A hallmark physiological response to estrogenic stimuli is the proliferation of cells *in vivo,* and this may promote tumor growth. This cell proliferation can also be mimicked *in vitro*. The E-screen uses established cell lines that are known to respond to estrogens and with it measures cell proliferation in response to increasing doses of the test compound. This assay is widely used and acknowledged as a reliable and valid test for a physiological response to estrogen action and can also discriminate agonists from antagonists [36].

The first step to evaluate the estrogenic activity of nemorosone by the E-screen assay was to construct the dose-proliferative response curve for E_2_ in MCF-7 BUS cells, and use it as a reference curve. The concentrations of 10^-6^M to 10^-11^M E_2_ were tested, being the 10^-8^ M the best concentration to induce cell proliferation (data not shown).

The growth of MCF-7 BUS cells was performed in order to ascertain the function of nemorosone as an estrogen agonists or as an estrogen antagonists. MCF-7 BUS cells were treated with benzophenone for 6 days, and cell growth was measured by the SRB method [27]. The effects on the proliferation of MCF-7 BUS cells of E_2_ and nemorosone are shown in Table 1. The results are expressed as PE and RPE %. The proliferative effects of the test compound relative to E_2_ (10^-8^M, 100%) are represented as the RPE (Relative Proliferative Effect). The benzophenone exhibited no estrogenic activity at any tested concentration.

**Table 1:** **Proliferative effects (PE) and relative proliferative effects (RPE%) of nemorosone (NM) and E**_**2 **_**according to the E-screen assay**

**Compound**	**Max PE**	**Max RPE%**
Positive control (E_2_)	3.44±0.57	100
NM 10^-9^M	1.02±0.01	0.8
NM 10^-8^M	1.09±0.08	3.82
NM 10^-7^M	1.03±0.02	1.07
NM 10^-6^M	1.05±0.03	1.87
NM 10^-5^M	1.10±0.08	3.94

The results of this assay indicated that the nemorosone was unable to induce a significant cell proliferation by comparison with the E_2_ (RPE = 100%) or more effectively than the solvent control (DMSO 0.01%).

**Figure 3:**
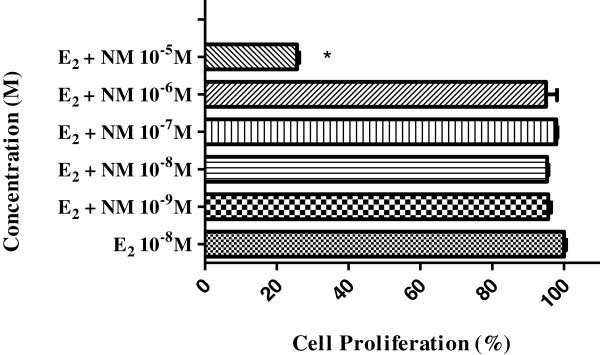
**Effects of nemorosone (NM) on MCF-7 BUS cell proliferation.** The cells were treated with E_2_ (10^-8^M) and nemorosone (10^-5^ to 10^-9^M) for 144 h. The SRB assay was conducted to measure cell proliferation. The inhibition E_2_-induced MCF-7 BUS (100% proliferation) by the nemorosone treatment is represented by cell proliferation in percentage. These results are expressed as the mean±SD of three separate experiments. Values significantly different from the E_2_ control are indicated by an asterisk (*p<0.05 by Student’s t-tests).

In order to determine whether the nemorosone induced antiestrogenic activity, MCF-7 BUS cells were treated with a combination of E_2_ (10^-8^M) and the test compound at various concentrations, as shown in Figure 3. The highest concentration (10^-5^M) reduced the cell proliferation induced by E_2_ in 75%. Since citotoxicity was not observed after the nemorosone treatments we suggested that the inhibitory effects of the compound on E_2_-induced cell proliferation must be related to antiestrogenic activity. The maximum proliferative response elicited by 10^-8^M E_2_ was inhibited significantly by 10^-5^M nemorosone.

Nowadays, there is a wide interest in phytoestrogens due to their potential health benefits in countering menopausal symptoms and in lowering incidence of hormone-dependent diseases including breast cancer and prostate cancer. The anticancer properties of phytoestrogens have been documented *in vivo* and *in vitro*, including inhibition of cell proliferation and angiogenesis [37].

Currently, the first-line agent for of ER*a*+breast cancer treatment is tamoxifen, [38] a selective ER modulator that antagonizes some *in vivo* effects of estrogens [39]; however, most tamoxifen-responsive breast cancer patients become tamoxifen resistant [40]. The second-line drug given to post-menopausal women with ER*a*+breast cancer is fulvestrant (ICI 182,780), a selective ER downregulator that completely abrogates estrogen-sensitive gene transcription. However, the resistance to fulvestrant is becoming a concern, too [40] . The limited effectiveness of current chemotherapeutic drugs underscores the importance of identifying novel targeted therapies with minimal side effects.

Nemorosone has been reported in recent years to be a potent cytotoxic agent particularly in aggressive cancer models characteristic for their highly chemoresistance. More interestingly, these compounds are less cytotoxic in normal, non-tumorigenic cells [3].

Popolo *et al.*[9], demonstrated that nemorosone exerted a concentration-dependent antiproliferative activity in MCF-7 by MTT assay. This effect seems to involve the nemorosone/ERα interaction, since the cytotoxic effect of nemorosone was significantly reduced by E_2_, significantly enhanced by ICI 182,780 and completely absent in MDA-MD-231 (a human breast cancer cell line ERα–) and in LNCaP (a human prostate carcinoma cell line ERα–/ERb+).

The majority of anticancer agents has DNA-damaging properties and affects not only the target-cells but also non-tumor cells. Their genotoxicity has been demonstrated in experimental models and in cancer patients treated with chemotherapy [41]. With the intention of in the future using nemorosone as adjuvant in breast cancer treatment, the comet assay was employed to evaluate DNA damage induced by the compound. This assay is widely regarded as a quick and reliable method of analyzing DNA damage in individual cells [42].

DNA fragmentation (%DNA in comet tail and olive tail moment) determined by the comet assay in the MCF-7 BUS and MCF10A cells, that was treated for 3 and 24 hours with nemorosone (10^-9^ to 10^-5^ M), showed no significant differences from the control cells (Tables 2 and 3). This indicates that the compound did not induce detectable DNA damage in these cells, detectable by this methodology.

**Table 2:** **Genotoxic activity expressed by the mean and the standard deviation (SD) of the *Olive Tail Moment *(OTM) and percentage DNA in comet tail (% DNA) in MCF-7 BUS cells treated with nemorosone for 3 and 24 hours and controls**

	**OTM**	**%DNA**	**OTM**	**%DNA**
	**Mean ± SD**	**Mean ± SD**	**Mean ± SD**	**Mean ± SD**
**Treatments**	** 3 hours**		** 24 hours**	
Control	1.56±2.00	8.17±6.56	1.69±3.83	3.65±7.45
DMSO	1.84±2.57	8.26±7.67	2.05±3.29	4.98±3.52
10^-9^M	1.23±1.88	7.75±8.32	1.52±1.92	4.47±6.00
10^-8^M	1.51±1.75	9.45±8.32	2.05±3.78	5.77±5.89
10^-7^M	1.35±1.47	6.17±7.98	2.35±1.98	6.17±5.12
10^-6^M	1.51±1.89	9.01±8.02	2.32±2.96	6.17±4.00
10^-5^M	1.11±1.73	7.67±7.98	0.94±1.60	2.48±6.76

Negative control: Culture Medium (DMEM).

DMSO: Solvent control (0.01%).

**Table 3:** **Genotoxic activity expressed by the mean and the standard deviation (SD) of the *Olive Tail Moment *(OTM) and percentage DNA in comet tail (% DNA) in MCF10A cells treated with nemorosone for 3 and 24 hours and controls**

	**OTM**	**%DNA**	**OTM**	**%DNA**
	**Mean ± SD**	**Mean ± SD**	**Mean ± SD**	**Mean ± SD**
**Treatments**	** 3 hours**		** 24 hours**	
Control	1.90±2.56	9.80±10.17	1.78±2.18	10.93±4.27
DMSO	2.27±1.25	9.92±8.99	2.53±2.07	15.27±4.78
10^-9^M	1.95±2.42	8.10±9.17	2.27±1.50	11.78±3.16
10^-8^M	1.64±2.52	8.31±9.70	1.84±0.29	13.04±5.25
10^-7^M	1.26±1.44	7.67±5.70	1.48±0.28	10.23±7.91
10^-6^M	1.63±2.07	8.23±8.79	2.44±3.62	13.46±4.52
10^-5^M	1.58±2.14	7.76±8.31	1.83±2.40	11.38±3.87

Negative control: Culture Medium (DMEM).

DMSO: Solvent control (0.01%).

Our results showed that nemorosone did not induce DNA damage using this method in the concentration range tested (10^-9^ to 10^-5^ M), neither in breast cancer cells MCF-7 BUS or in normal breast cells. Therefore, it is reasonable to hope that nemorosone could be a promising adjuvant for ER antagonists in ERα + breast cancer prevention and (or) treatment.

## Conclusion

The present work demonstrates that nemorosone presents no genotoxic property and posses antiestrogenic activity, reducing the cell proliferation induced by E_2_. Further studies are required in order to establish the introduction of nemorosone on breast cancer treatment.

## Abbreviations

ER: Estrogen receptor; RYA: Recombinant yeast assay; DMSO: Dimethyl sulfoxide; NM: Nemorosone; SRB: Sulforhodamine B; PE: Proliferative effect; RPE: Relative proliferative effect; FCS: Fetal calf serum; EDTA: Ethylenediaminetetraacetic; OTM: Olive tail moment; %DNA: Percentage of DNA in the tail

## Competing interest

There was no competing interest. The authors alone are responsible for the content and writing of the paper. All the authors have approved the final article.

## Authors’ contributions

Camargo, M.S. designed and performed the experiments, interpreted the results and drafted the manuscript. Prieto, A.M. designed and performed the comet assay. Resende, F.A. and Boldrin, P.K. participated in the RYA experiments. Cardoso, CRP; Fernández, M.F.; Molina-Molina, J.M. and Olea, N. designed and performed the E-screen assay. Vilegas, W and Cuesta-Rubio, O. isolated the nemorosone. Varanda, E.A. critically read the manuscript and participated in revision of the manuscript. All authors have read and approved the final manuscript.

## Pre-publication history

The pre-publication history for this paper can be accessed here:

http://www.biomedcentral.com/1472-6882/13/201/prepub
